# LVEF by Multigated Acquisition Scan Compared to Other Imaging Modalities in Cardio-Oncology: a Systematic Review

**DOI:** 10.1007/s11897-022-00544-3

**Published:** 2022-03-30

**Authors:** Markella I. Printezi, Laura I. E. Yousif, Janine A. M. Kamphuis, Linda W. van Laake, Maarten J. Cramer, Monique G. G. Hobbelink, Folkert W. Asselbergs, Arco J. Teske

**Affiliations:** 1 Department of Cardiology, Division of Heart and Lungs, University Medical Center Utrecht, Utrecht University, Room number F02.318, P.O. Box 85500, 3508 GA Utrecht, The Netherlands; 2 Graduate School of Life Sciences, University Medical Center Utrecht, Utrecht University, Utrecht, The Netherlands; 3 Department of Radiology and Nuclear Medicine, University Medical Center Utrecht, Utrecht University, Utrecht, The Netherlands; 4 Health Data Research UK and Institute of Health Informatics, University College London, London, UK; 5 Institute of Cardiovascular Science, Faculty of Population Health Sciences, University College London, London, UK

**Keywords:** Cardio-oncology, Cardiotoxicity, Multigated acquisition scan, Cardiac magnetic resonance imaging, Echocardiography, Left ventricular ejection fraction

## Abstract

**Purpose of Review:**

The prevalence of cancer therapy-related cardiac dysfunction (CTRCD) is increasing due to improved cancer survival. Serial monitoring of cardiac function is essential to detect CTRCD, guiding timely intervention strategies. Multigated radionuclide angiography (MUGA) has been the main screening tool using left ventricular ejection fraction (LVEF) to monitor cardiac dysfunction. However, transthoracic echocardiography (TTE) and cardiac magnetic resonance imaging (CMR) may be more suitable for serial assessment. We aimed to assess the concordance between different non-radiating imaging modalities with MUGA to determine whether they can be used interchangeably.

**Recent Findings:**

In order to identify relevant studies, a PubMed search was performed. We included cross-sectional studies comparing MUGA LVEF to that of 2D TTE, 3D TTE, and CMR. From 470 articles, 22 were selected, comprising 1017 patients in total. Among others, this included three 3D TTE, seven 2D harmonic TTE + contrast (2DHC), and seven CMR comparisons. The correlations and Bland-Altman limits of agreement varied for CMR but were stronger for 3D TTE and 2DHC.

**Summary:**

Our findings suggest that MUGA and CMR should not be used interchangeably whereas 3D TTE and 2DHC are appropriate alternatives following an initial MUGA scan. We propose a multimodality diagnostic imaging strategy for LVEF monitoring in patients undergoing cancer treatment.

**Supplementary Information:**

The online version contains supplementary material available at 10.1007/s11897-022-00544-3.

## Introduction

Cancer treatment has become more effective over the past decades, resulting in increasing numbers of cancer survivors globally [1]. Unfortunately, cancer treatment can lead to cancer therapy-related cardiac dysfunction (CTRCD) due to its direct and indirect cardiotoxic effects, such as ischemia, hypertension, and vascular and metabolic dysregulation [2]. The resulting cardiomyocyte damage and dysfunction can in turn lead to congestive heart failure [3]. In this manner, cardiovascular complications resulting from cancer treatment contribute greatly to the global burden of cardiovascular disease and represent a leading cause of death in cancer survivors [1, 3–5]. While the progressive decline in cardiac function due to cancer treatment can occur up to 20 years after treatment, research has shown that it can be prevented or ameliorated through early monitoring, continued cardiac surveillance during and after treatment, and timely intervention [6].

According to the European Society of Cardiology (ESC), the optimal parameter of cardiac function assessment in the field of cardio-oncology is left ventricular ejection fraction (LVEF). The ESC defines CTRCD as a decrease in LVEF by more than 10 percentage-points below the value of 53%, which is the normal reference value of two-dimensional echocardiography (2D TTE), or below 50%, which is the lower limit of normal for multigated acquisition (MUGA) scan [7, 8]. Timely interruption of cancer treatment and/or early initiation of cardioprotective treatment is often necessary to impede further LVEF decline and optimize the possibility of LVEF recovery in asymptomatic cardiac dysfunction [3, 9]. Therefore, serial cardiac monitoring using the optimal method of imaging is crucial.

MUGA became popular in the 1970s when researchers were trying to identify patients with a decline in LVEF prior to heart failure symptoms [7]. To date, the MUGA scan is being used in clinical settings for LVEF monitoring due to its high reproducibility, low variability, and few technical limitations. Two main disadvantages are its lack in ability to evaluate structural and functional abnormalities beyond LVEF and its exposure of patients to additional ionizing radiation [7, 10]. The effect of cumulative radiation is feared, especially so for the long-term consequences it may have for young patients [4]. Consequently, implementation of an alternative imaging modality is desired.

The ESC and EACVI advise to first and foremost use echocardiography for the serial assessment of LVEF in patients at risk for CTRCD [7, 8]. Three-dimensional echocardiography (3D TTE) is preferred as its lack of reliance on geometrical assumptions and its ability to provide a scan of the entire left ventricular cavity yield highly accurate and reproducible results [11, 12]. Alternatively, cardiac magnetic resonance (CMR) imaging is considered the current gold standard for left and right ventricular volumes and their function due to its clear and reliable measurements [7, 13].

Within cardio-oncological screening, multiple cardiac imaging modalities are interchangeably used due to an unexpected necessity of alternative imaging modalities (e.g., in patients where TTE becomes unsuitable for LVEF follow-up after a left-sided mastectomy). The ability of different imaging modalities to yield accurate and, most importantly, comparable results remains to be fully determined. This is relevant for improving patient care as changes in cancer treatment based on inaccurate LVEF evaluations can have severe consequences [6]. Therefore, this systematic review aims to assess the interchangeability of MUGA with other cardiac imaging modalities.

## Methods

This systematic review was performed in accordance with the PRISMA statement (Preferred Reporting Items for Systematic Reviews and Meta-Analyses) [14]. A comprehensive PubMed search was performed and updated on January 17, 2022 using the search query listed in Supplemental Table 1. This list consists of synonyms for the MUGA scan, each of the reference tests and LVEF. Additionally, the following filters were applied: humans, adults (≥18 years old), and English language. The reference lists of all selected papers were hand searched and relevant studies were included.

This systematic review includes cross-sectional studies comparing LVEF measurements of the MUGA scan with at least one of the appropriate reference tests: contrast angiography, 2D TTE, 3D TTE, CMR, and thermodilution (TD). For 2D TTE, reporting of the biplane method of disks, or modified Simpson’s rule, was a requirement. Papers assessing LVEF by visual assessment only were excluded. Additionally, since harmonic 2D TTE provides images of superior quality to fundamental imaging, only harmonic 2D TTE was used for the analysis in studies reporting both harmonic and fundamental images [15]. However, if the study only researched fundamental 2D TTE, it was still included. For the final analysis, we considered the different echocardiographic techniques (fundamental, harmonic, harmonic with contrast, and 3D) as separate [16]. We excluded ionizing imaging modalities, such as computed tomography (CT) and single-positron emission computed tomography (SPECT). No distinctions were made between different MUGA techniques, such as first-pass MUGA and planar MUGA [17]. Articles were included based on their abstracts and full text availability as well as the following inclusion criteria: English language, human population, adult population (≥18 years), and a comparison between MUGA with at least one of the mentioned reference tests. Afterward, full texts of the remaining articles were read, implementing the exclusion criteria to narrow down the search to the most relevant articles. Exclusion criteria were MUGA and reference test performed more than 30 days apart, unclear or invalid correlation analysis method, and no LVEF measurement reported. The methodological quality of each article was assessed using the National Heart, Lung and Blood Institute’s Quality Assessment Tool for Observational Cohort and Cross-Sectional Studies and all studies of poor quality were excluded [18].

The following parameters were extracted from the included studies: study design (prospective/retrospective), population size, female to male ratio, mean age, main cardiac disease, reference test(s), correlation coefficient(s), Bland-Altman 95% limits of agreement (LOAs), and the number of days between the reference test(s) and MUGA. In case multiple correlation values were reported in a single study (e.g., measurements done at different time points), this was transformed into a mean value if possible — likewise for Bland-Altman values. As a rule of thumb for Pearson correlation coefficient analysis, a value of 0.0–0.3 was considered to reflect negligible, 0.3–0.5 low, 0.5–0.7 moderate, 0.7–0.9 high, and 0.9–1.0 very high correlation. Since the definition of CTRCD indicates a change in more than 10 percentage-point change of LVEF, LOAs over 10% either way of the line were considered to represent an unsuitable imaging modality to use interchangeably with MUGA [19]. Percentages were calculated for the study population characteristics and the correlation coefficients and Bland-Altman values were plotted to compare between the various studies and modalities.

## Results

**Fig. 1:**
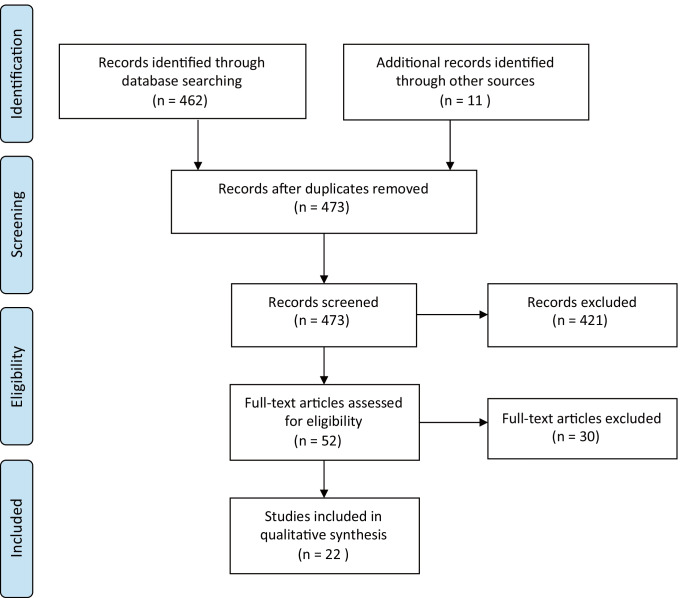
PRISMA flow chart of the article selection process

Our search yielded a total of 473 articles, as illustrated in the PRISMA flow chart depicted in Figure 1 [20]. Of the 473 articles, 142 were excluded based on language, full text availability, or population age. Through title and abstract screening, 279 articles were excluded based on incomplete agreement with our predefined inclusion criteria. Of the 52 articles assessed on full text, 30 were excluded based on exclusion criteria or missing inclusion criteria. The remaining 22 articles were included in this study and data was extracted for qualitative analysis.

Table 1 provides an overview of the characteristics of the included publications. In total, the studies comprised 1017 patients with a mean age of 56 years, and the male sex was predominant. A prospective study design was used in 18 out of 22 studies. Of the included studies, 41% was conducted between 1984 and 2000, 41% between 2000 and 2010, and 18% between 2010 and 2020. In total, 32 comparisons were made, with 2DHC and CMR being the most common reference tests (22% both). However, when pooling all different 2D TTE methods, this made up 53% of the comparisons. Furthermore, three comparisons were made with 3D TTE, four with contrast angiography, and one with TD.

**Table 1:** Overview of selected studies

Author, year of publication	Study design (P/R)	Sample size, female:male	Age (mean, years)	Main cardiac disease	Reference test	Days between tests	*r*	BA LOAs	BA mean bias
Vandenbossche et al. (1984) [35]	R	*n* = 20, 19:1	46	Chronic aortic regurgitation	2DF	Same day	0.81	N/A	N/A
Høilund-Carlsen et al. (1984) [36]	P	*n* = 12, 2:10	55	Previous MI	TD	Same day	0.95	N/A	N/A
Verani et al. (1985) [37]	P	*n* = 29, N/A	56	CAD	A	≤2	0.85	N/A	N/A
Corbett et al. (1985) [38]	P	*n* = 39, 13:24	31	CAD	A	≤10	0.88	N/A	N/A
Gaudio et al. (1991) [26]	P	*n* = 32, 9:23	49	CMP	CMR	Same day	0.92	−4, 7.5	1.8
Naik et al. (1995) [39]	P	*n* = 25, 10:15	68	Previous MI	2DF	≤10	0.93	−11.7, 11.5	−0.1
					A	≤10	0.87	N/A	N/A
Chin et al. (1997) [17]	P	*n* = 18, 7:11	46	Pot. LV dysf.	CMR	Same day	0.94	N/A	N/A
Jensen-Urstad et al. (1998) [40]	P	*n* = 107, 29:78	64	AMI	2DF	Same day	0.45	−17.5, 17.5	0
Nosir et al. (1998) [27]	P	*n* = 41, 12:29	51	Previous MI	3D	≤4	0.99	−6.7, 6.9	0.1
					2DF	≤4	0.97	−8.3, 9.7	0.7
Nahar et al. (2000) [31]	P	*n* = 50, 19:31	47	Pot. LV dysf.	2DH	Same day	0.84	−7.5, 10.8	1.7
					2DHC	Same day	0.95	−6, 4.2	−0.9
Yu et al. (2000) [33]	P	*n* = 51, 19:23	57	Hypertension	2DH	Same day	0.89	−15, 17	1
					2DHC	Same day	0.97	−7, 7	0
Takuma et al. (2001) [28]	P	*n* = 43, 15:28	57	Heart transplant	2DF	Same day	0.85	−15.9, 9.1	−3.38
					3D	Same day	0.87	−15.4, 11.3	−2.01
Dias et al. (2001) [41]	P	*n* = 62, 19:43	59	Pot. LV dysf.	2DHC	Same day	0.97	−6, 4.9	−0.6
Bellenger et al. (2002) [23]	P	*n* = 12, 3:9	51	Heart transplant	A	≤11	0.4	−30, 15	−7.4
					CMR	≤11	0.3	−11, 17	3
Yu et al. (2003) [32]	P	*n* = 51, 21:30	62	CAD	2DHC	Same day	0.95	−13, 11	−1
					2DHC	Same day	0.96	−11, 10	−0.5
Mohan et al. (2004) [42]	P	*n* = 16, 6:10	60	Previous MI	2DH	≤7	0.71	−21.5, 10.5	0.5
Galasko et al. (2004) [43]	R	*n* = 86, 14:72	50	AMI	2DF	Same day	0.9	−10.2, 13	1.4
Bezante et al. (2005) [44]	P	*n* = 33, N/A	64	CAD	2DHC	≤7	0.82	N/A	N/A
					2DH	≤7	0.74	N/A	N/A
					2DHC	≤7	0.89	N/A	N/A
Walker et al. (2010) [25]	P	*n* = 50, 0:50	52	Pot. cardiotox.	3D	≤7	0.89	−4, 5.2	0.6
					CMR	≤7	0.91	−4.8, 3.8	−0.5
Huang et al. (2017) [21]	R	*n* = 75, 31:44	58	Pot. CMP	CMR	≤30	0.63	−19.4, 16.5	−1.5
Kotha et al. (2018) [22]	R	*n* = 124, 29:95	64	Arrhythmias	CMR	≤30	0.86	−12.1, 11.4	0.4
Dhir et al. (2019) [24]	P	*n* = 41, 0:41	51	Pot. cardiotox.	CMR	≤14	0.39	−15.4, 9.5	−3

BA LOAs and mean bias both depict MUGA LVEF subtracted by the reference test LVEF

*2DF* two-dimensional fundamental echocardiography, *2DH* two-dimensional harmonic echocardiography, *2DHC* two-dimensional harmonic echocardiography with contrast, *3D* three-dimensional echocardiography, *A* angiography, *AMI* acute myocardial infarction, *BA* Bland-Altman, *cardiotox* cardiotoxicity, *CAD* coronary artery disease, *CMP* cardiomyopathy, *CMR* cardiac magnetic resonance imaging, *dysf.* dysfunction, *LOAs* limits of agreement of left ventricular ejection fraction (LVEF), *LV* left ventricle, *MI* myocardial infarction, *N/A* information not available, *P* prospective, *PE* pulmonary embolism, *pot.* potential, *R* retrospective, correlation of LVEFs, and *TD* thermodilution

**Fig. 2:**
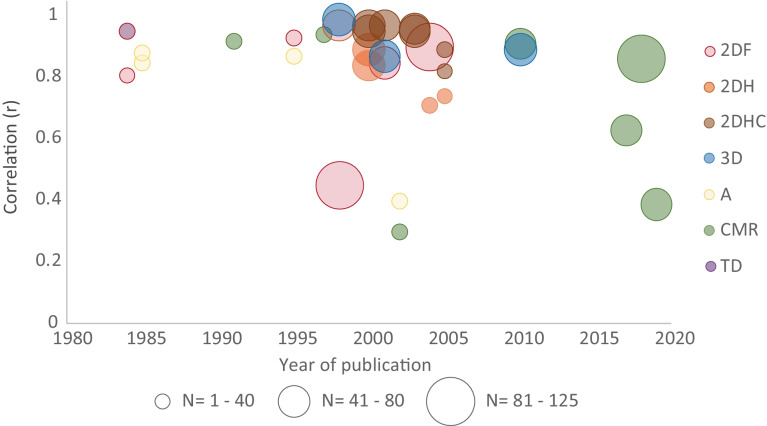
Bubble chart depicting correlations between MUGA and other imaging modalities. Most represent the Pearson correlation coefficient, while some studies reported Lin’s concordance (asterisk) or a correlation coefficient from linear regression analysis (dagger). The bubbles represent the correlation between the mentioned tests and MUGA over time, while factoring in the number of participants included (bubble size). *2DF* two-dimensional fundamental echocardiography (red), *2DH* two-dimensional harmonic echocardiography (orange), *2DHC* two-dimensional harmonic echocardiography with contrast (brown), *3D* three-dimensional echocardiography (blue), *A* angiography (yellow), *CMR* cardiac magnetic resonance imaging (green), and *TD* thermodilution (purple)

An overview of the correlation coefficients for all comparisons is shown in Figure 2. 2DH shows high correlation overall, with a slight decrease in the latest two studies. 2DHC particularly provides high correlation with MUGA scan throughout the course of time, with five very high and two high correlations. All 3D TTEs showed high correlation values as well. Figure 2 also illustrates varying correlations between MUGA and CMR from 2010 onward. TD was compared once, using cardiac output instead of LVEF, and was reported to correlate very strongly with MUGA results.

**Fig. 3:**
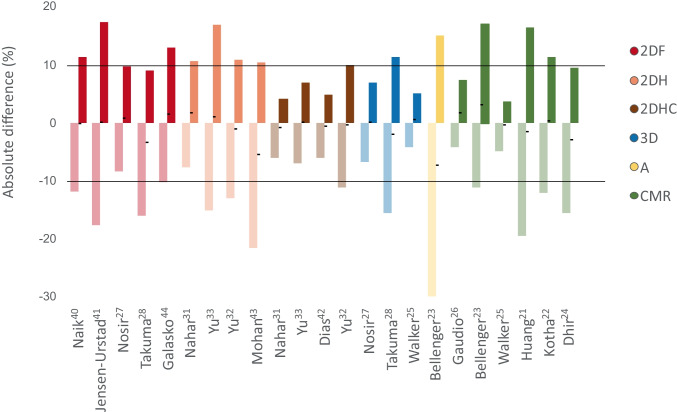
Overview of Bland-Altman 95% limits of agreement. Mean bias is depicted as a black horizontal stripe, the 95% LOA by the colored bars, and the predefined clinical acceptable limit of 10% left ventricular ejection fraction (LVEF) differences by the horizontal lines at ±10% LVEF. *2DF* two-dimensional fundamental echocardiography (red), *2DH* two-dimensional harmonic echocardiography (orange), *2DHC* two-dimensional harmonic echocardiography with contrast (brown), *3D* three-dimensional echocardiography (blue), *A* angiography (yellow), and *CMR* cardiac magnetic resonance imaging (green)

Unfortunately, not all publications reported Bland-Altman LOAs. Of 22 included publications, 16 reported 23 comparisons using Bland-Altman. Figure 3 illustrates that only eight publications had LOAs within the predefined ±10% range; this was predominantly seen for 2DHC (three out of four) and for 3D TTE (two out of three). Almost all 2DF (four out of five) and all the 2DH and angiography comparisons had weak agreements. Interestingly, CMR showed poor agreement with MUGA, with broad LOAs that exceeded the ±10% range in four out of six studies. In particular, the three most recent studies showed weak agreement, with LOAs of −19.4 to 16.5% for Huang et al., −12.1 to 11.4% for Kotha et al., and −15.4 to 9.5% for Dhir et al. [21, 22, 24]. Two older studies by Walker et al. and Gaudio et al. indicated appropriate LOAs of −4.8 to 3.8% and −4 to 7.5%, respectively [25, 26]. However, their cumulative number of subjects (*n* = 82) accounted for only 23% of the total.

## Discussion

Standardized diagnostic protocols for serial monitoring of LVEF are of crucial importance in cardio-oncological care [2, 7, 9]. As many different non-invasive cardiac diagnostic modalities have evolved, determining their interchangeability is essential. MUGA provides limited cardiac information and contributes to cumulative radiation exposure. Therefore, it has become clear that MUGA should be replaced as the primary method of LVEF monitoring, despite its excellent reproducibility. Therefore, we set out to compare the concordance of LVEF measurements between MUGA and other diagnostic tests to determine which imaging modality should be chosen when local institution guidelines are to be changed from MUGA to an alternative. Additionally, the findings of our systematic analysis will aid in determining the position of MUGA in an imaging strategy when suitable alternatives must be available for echocardiography (e.g., due to poor image quality) and CMR (e.g., due to claustrophobia). Our first main finding was that only 3D TTE and 2DHC correlate well with MUGA and show variability within our pre-defined LOA range of ±10% LVEF units. To our surprise, our second finding revealed that CMR and MUGA do not appear to be interchangeable since most studies (four out of six) reported LOAs exceeding ±10%. Thus, this variability could potentially result in an incorrect diagnosis of CTRCD when these two techniques are used within a single patient.

3D TTE was unfortunately not compared with MUGA as frequently as 2DHC and CMR. Even though no definitive conclusions can be drawn from only three articles (*n* = 134), the strong correlations and narrow LOAs reported by Walker et al. and Nosir et al. demonstrate that 3D TTE LVEF measurements are highly comparable to MUGA [25, 27]. Both publications had two independent interpreters that blindly evaluated LVEF at separate occasions to minimize observer bias. The third 3D TTE study by Takuma et al. had a weak agreement (despite a correlation of 0.87) that may be explained by their use of an outdated post processing technique [28]. These findings are in line with the recommendation of the EACVI to use 3D TTE as the primary method for serial LVEF monitoring [7, 25, 29].

CMR had correlation coefficients ranging from low to very high and variable LOAs as seen in Figures 2 and 3. In contrast to our expectations, our findings reveal a generally poor agreement between MUGA and CMR. Namely, out of six comparisons with correlation coefficients and Bland-Altman LOAs, four (72% of patients) show poor concordance between MUGA and CMR, strongly suggesting the two are not to be used interchangeably. Interestingly, when comparing CMR and 3D TTE, Walker et al. observed a strong correlation (0.93) and appropriate LOAs of −6.5 to 3% [30]. This may implicate that the recommendation to use CMR as a complementary method only holds true when the primary evaluation was performed with 3D TTE and not MUGA [7, 10]. We lack a good explanation why, in this study, CMR and 3D TTE have an excellent agreement, while a discrepancy exists between the good agreement of MUGA and 3D TTE and the poor agreement between MUGA and CMR.

Figures 2 and 3 illustrate comparable 2DF and 2DH results whereas the superiority of 2DHC is obvious. Our data show that, with seven studies showing very high correlation and three out of four studies with appropriate agreement with MUGA results, 2DHC is the most feasible MUGA replacement when 3D TTE is unavailable [8, 12, 31, 32]. While the two most recent correlation coefficients suggest a slight decline in correlation, the population size of these studies is quite small (33% of patients) which might have affected the results. Normally, contrast administration is advised when images are not sufficiently clear, as it enhances the LV borders. The accuracy and strong correlation with MUGA support this recommendation and should thus be considered in selected cases [10, 33, 34].

Notably, some of the included studies investigated LVEF measurements across different imaging modalities in patients with (possible) cardiotoxicity. As serial monitoring is essential to detect early cardiac dysfunction, these studies compared methods with MUGA over several time points. For example, Walker et al. conducted a long-term research with serial assessments on patients receiving anthracyclines or trastuzumab for breast cancer [25]. They performed real-time 3D TTE, CMR, and MUGA tests at baseline, 6, and 12 months after initiation of trastuzumab and found that 3D TTE was a feasible, accurate, and reproducible alternative for serial monitoring of LVEF. Dhir et al. as well came to the resolution that CMR and MUGA were not interchangeable and serial monitoring should be done with one of the two methods exclusively [24].

Only a limited number of articles were available comparing methods performed on patients undergoing cancer treatment. Instead, most of the publications involved patients with other existing or suspected cardiac problems. Furthermore, technological improvements during the studied period do not allow for any definite conclusions based on the older comparative studies. For example, no study compared 3D TTE with MUGA after 2010, while many technological improvements have occurred that could presumably significantly improve the compatibility between the two methods. Finally, it should be taken into consideration that only two studies included over 100 patients and 46% of the studies had less than 40 patients.

## Clinical perspectives

**Fig. 4:**
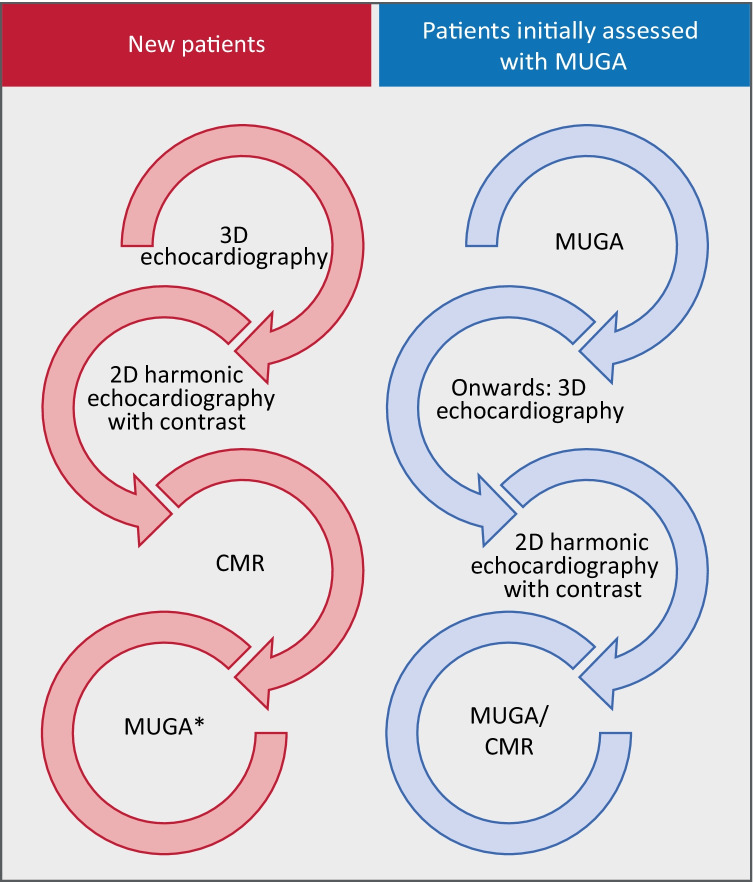
Central illustration: flow diagram of proposed imaging strategy for patients at risk for cancer therapy-related cardiac dysfunction. Preferred modality ranges from top to bottom, from most to least preferred. *2D* two-dimensional, *3D* three-dimensional, and *MUGA* multigated acquisition scan. *LVEF obtained by MUGA after CMR monitoring should be interpreted cautiously, since these modalities show poor agreement with one another

Our findings should be interpreted as clinical recommendations regarding imaging modalities which can be used interchangeably with MUGA to ensure adequate identification of cardiotoxic severity and minimize interpatient variability. Our proposed diagnostic imaging strategy for serial LVEF monitoring in cancer patients at risk for CTRCD, with respect to MUGA, is illustrated in Figure 4. At baseline, 3D TTE should be used as the initial method of LVEF evaluation as it was found to be the most reproducible echocardiographic technique for LVEF measurement over a 1-year follow-up in patients undergoing chemotherapy [11]. Furthermore, it has lower costs and is more widely available than CMR. If unavailable or images of better quality are needed, 2DHC should be performed. In case of unsuitable LVEF calculation by 2DHC, CMR has the preference due to its high correlation with 3D TTE and lack of radiation that MUGA bears [30]. However, MUGA can be used if CMR is unavailable [25]. For patients who have been initially assessed with MUGA, a switch to 3D TTE is advised to avoid cumulative radiation exposure. Alternatively, 2DHC can be used. If the images are unclear, it is more sensible to revert to MUGA, as opposed to CMR, as that would concur most with the initial images taken and thus represent a more accurate monitoring of ejection fraction within a single patient. In patients with unexplained cardiac dysfunction, in those with possible concomitant ischemia, or in those where advanced assessment of tissue characteristics is warranted (e.g., suspicion of myocarditis), the role of CMR is unquestioned and should be considered with a very low threshold to complement the findings of ultrasound in order to better understand the underlying cardiac mechanism in the cancer patient guiding therapeutic decision making [45].

## Conclusion

In conclusion, when substituting LVEF assessing imaging modalities for MUGA, the findings of this systematic review support the use of echocardiography (3D in particular), while CMR should be used cautiously due to its large variability with MUGA. For serial monitoring of LVEF in patients with (possible) CTRCD, 3D TTE, or alternatively 2DHC, may be the best initial cardiac imaging method.

## Supplementary Information

Below is the link to the electronic supplementary material.

Supplementary file1 (DOCX 16 kb)

## Data Availability

The data that support the findings of this systematic review are available through the original manuscripts to which we refer. No new data were generated in support of this research.
