# Supplementary material for: LVEF by Multigated Acquisition Scan Compared to Other Imaging Modalities in Cardio-Oncology: a Systematic Review
Source: Curr Heart Fail Rep. 2022 Mar 30;19(3):136–45. doi: 10.1007/s11897-022-00544-3 (PMC9177497; doi:10.1007/s11897-022-00544-3)
Supplement: Supplementary file 1 — Supplementary file1 (DOCX 16 kb) [file 11897_2022_544_MOESM1_ESM.docx]

**Supplemental Appendix**

Contents

Supplemental Table 1. Search Query. Page 2.

**Supplemental Table 1. Search query**

(MUGA[Title/Abstract] OR ''multigated acquisition''[Title/Abstract] OR ''multi-gated acquisition''[Title/Abstract] OR ''multiple gated acquisition''[Title/Abstract] OR ''multiple- gated acquisition''[Title/Abstract] OR ''multiple-gated blood pool angiography''[Title/Abstract] OR ''multigated blood pool angiography''[Title/Abstract] OR ''multi-gated blood pool angiography''[Title/Abstract] OR ''multiple gated cardiac blood pool''[Title/Abstract] OR ''multigated cardiac blood pool imaging''[Title/Abstract] OR ''gated blood pool imaging''[Title/Abstract] OR ''blood pool scan''[Title/Abstract] OR ''gated blood- pool''[Title/Abstract] OR ''gated blood pool''[Title/Abstract] OR ''radionuclide imaging''[Title/Abstract] OR ''multigated radionuclide angiography''[Title/Abstract] OR ''equilibrium radionuclide angiography''[Title/Abstract] OR ''tomographic radionuclide angiography''[Title/Abstract] OR ''tomographic radionuclide ventriculography''[Title/Abstract] OR ''equilibrium radionuclide ventriculography''[Title/Abstract] OR ''blood pool scintigraphy''[Title/Abstract]) AND (''left ventricular ejection fraction''[Title/Abstract] OR ''ejection fraction''[Title/Abstract] OR ''ventricular ejection''[Title/Abstract] OR ''ventricular fraction''[Title/Abstract] OR ''ventricular function''[Title/Abstract] OR ''systolic function''[Title/Abstract] OR ''left ventricular function''[Title/Abstract] OR ''ventricular outflow''[Title/Abstract] OR ''heart function''[Title/Abstract] OR '' cardiac function''[Title/Abstract] OR ''cardiac output''[Title/Abstract] OR ''stroke volume''[Title/Abstract] OR ''end diastolic volume''[Title/Abstract] OR ''end systolic volume''[Title/Abstract] OR ''left ventricular volume''[Title/Abstract]) AND (''magnetic resonance''[Title/Abstract] OR ''magnetic resonance imaging''[Title/Abstract] OR ''proton spin tomography''[Title/Abstract] OR ''cardiac MRI''[Title/Abstract] OR ''cardiac magnetic resonance imaging''[Title/Abstract] OR ''3D echocardiography''[Title/Abstract] OR ''2D echocardiography''[Title/Abstract] OR ''echocardiography''[Title/Abstract] OR ''sonography''[Title/Abstract] OR ''angiography''[Title/Abstract] OR ''swan-ganz''[Title/Abstract] OR ''swan ganz''[Title/Abstract] OR pulmonary artery catheterization''[Title/Abstract] OR ''computed tomographic angiography''[Title/Abstract] OR ''computed assisted tomography''[Title/Abstract] OR ''computed angiography''[Title/Abstract] OR ''ultrasound''[Title/Abstract]) AND (english[LA]) AND (humans[MeSH])
